# Comparative genomic analyses of *Mycoplasma hyopneumoniae* pathogenic 168 strain and its high-passaged attenuated strain

**DOI:** 10.1186/1471-2164-14-80

**Published:** 2013-02-05

**Authors:** Wei Liu, Shaobo Xiao, Mao Li, Shaohua Guo, Sha Li, Rui Luo, Zhixin Feng, Bin Li, Zhemin Zhou, Guoqing Shao, Huanchun Chen, Liurong Fang

**Affiliations:** 1 Division of Animal Infectious Diseases, State Key Laboratory of Agricultural Microbiology, College of Veterinary Medicine, Huazhong Agricultural University, Wuhan, 430070, People’s Republic of China; 2 Institute of Veterinary Medicine, Jiangsu Academy of Agricultural Sciences, Nanjing, 210014, People’s Republic of China; 3 Environmental Research Institute, University College Cork, Cork, Ireland

**Keywords:** *Mycoplasma hyopneumoniae*, Genetic variation, Virulence attenuation, Sequence analysis, Repetitive sequences, Virulence factors

## Abstract

**Background:**

*Mycoplasma hyopneumoniae* is the causative agent of porcine enzootic pneumonia (EP), a mild, chronic pneumonia of swine. Despite presenting with low direct mortality, EP is responsible for major economic losses in the pig industry. To identify the virulence-associated determinants of *M. hyopneumoniae*, we determined the whole genome sequence of *M. hyopneumoniae* strain 168 and its attenuated high-passage strain 168-L and carried out comparative genomic analyses.

**Results:**

We performed the first comprehensive analysis of *M. hyopneumonia*e strain 168 and its attenuated strain and made a preliminary survey of coding sequences (CDSs) that may be related to virulence. The 168-L genome has a highly similar gene content and order to that of 168, but is 4,483 bp smaller because there are 60 insertions and 43 deletions in 168-L. Besides these indels, 227 single nucleotide variations (SNVs) were identified. We further investigated the variants that affected CDSs, and compared them to reported virulence determinants. Notably, almost all of the reported virulence determinants are included in these variants affected CDSs. In addition to variations previously described in mycoplasma adhesins (P97, P102, P146, P159, P216, and LppT), cell envelope proteins (P95), cell surface antigens (P36), secreted proteins and chaperone protein (*DnaK*), mutations in genes related to metabolism and growth may also contribute to the attenuated virulence in 168-L. Furthermore, many mutations were located in the previously described repeat motif, which may be of primary importance for virulence.

**Conclusions:**

We studied the virulence attenuation mechanism of *M. hyopneumoniae* by comparative genomic analysis of virulent strain 168 and its attenuated high-passage strain 168-L. Our findings provide a preliminary survey of CDSs that may be related to virulence. While these include reported virulence-related genes, other novel virulence determinants were also detected. This new information will form the foundation of future investigations into the pathogenesis of *M. hyopneumoniae* and facilitate the design of new vaccines.

## Background

*Mycoplasma hyopneumoniae* causes porcine enzootic pneumonia, which is a mild, chronic pneumonia of swine [1]. This highly infectious organism has a worldwide distribution. The primary mycoplasmal infection often becomes complicated by secondary bacterial and viral infections [2], resulting in more severe lung lesions and production losses. Relative control has been achieved through active vaccination programs, but porcine enzootic pneumonia continues to be a major economic problem in the swine industry. While progress has been made in understanding the molecular basis of some *Mycoplasma* diseases [3], advances in *M. hyopneumoniae* research have been hampered by its fastidious growth condition and the lack of genetic tools and transformation protocols. To date, few virulence determinants or virulence-associated determinants have been identified. Attachment to the respiratory epithelium is a prerequisite for host colonization and is mediated by the membrane protein P97 [4]. This protein is located on the outer membrane surface, and its role in adherence has been firmly established. The general region of P97 that mediates adherence to swine cilia is thought to be the R1 region, near the C-terminus of the protein [5]. To bind cilia, a minimum of eight tandem copies of the pentapeptide sequence (AAKPV/E) in R1 are required [5]. Although the function of R2 *in vivo* is unknown, both it and R1 are required to bind heparin [6]. The P97 genes of *M. hyopneumoniae* strains 7448, 232, and J code for proteins with 10, 15, and 9 of the previously described R1 repeating units (AAKPV/E), respectively; all three strains had more than the minimum number of tandem copies (8 tandem copies) required for cilium binding [7]. Moreover, monoclonal antibodies F1B6 and F2G5, which both react predominantly with P97 [4,5], only partially block adherence of *M. hyopneumoniae* to receptors on epithelial cell cilia [8]. These observations indicate that molecules other than P97 play a role in facilitating adherence of *M. hyopneumoniae* to swine cilia. Comparative transcriptomic and proteomic studies are also performed to study transcriptional changes that occur during disease and investigate differentially expressed proteins in pathogenic and non-pathogenic strains [9-11]. Several *M. hyopneumoniae* proteins, including immunodominant proteins (P36 [12], P46 [13], and P65 [14]), adhesin-related proteins (P102 [15], P146 [16], P159 [17], P216 [18], and LppT [16]), and a 54-kDa cytotoxic factor [19], have been characterized; however, the biological functions of these proteins in pathogenesis are not well understood.

Comparative genomic analysis has previously revealed mechanisms of *M. hyopneumoniae* pathogenicity [7] and predicted unidentified virulence factors, including genes involved in secretion and/or traffic between host and pathogen cells, or with evasion and/or modulation of the host immune system [20,21]. In 2005, Vasconcelos *et al.* sequenced a pathogenic and a non-pathogenic strain of *M. hyopneumoniae* and performed a comparative genomics approach to identify putative virulence genes [7]. They identified various CDSs that could be considered candidate virulence genes, including cilium adhesin homologs, lipoproteins, and other components which might contribute to virulence [7]. However, comparative genomic analysis of a virulent *M. hyopneumoniae* strain versus its attenuated strain is lacking.

The need to control the spread of *M. hyopneumoniae* prompted the development of live attenuated vaccine strains. *M. hyopneumoniae* strain 168-L has been extensively used as vaccine against *M. hyopneumoniae* in China [22,23]. This attenuated vaccine strain is derived from the virulent parent strain 168. Strain 168 was originally isolated in 1974, from an Er-hua-nian pig (a Chinese local breed very sensitive to *M. hyopneumoniae*) with typical clinical and pathogenic characteristics of mycoplasmal pneumonia of swine (MPS) [24]. This field strain was gradually attenuated by more than 300 continuous passages through KM2 cell-free medium (a modified Friis medium) and the 380th passage was named strain 168-L. Currently, the genetic basis for the attenuation of virulence in 168-L is poorly understood.

To gain new insight into the components that contribute to virulence and the mechanisms by which *M. hyopneumoniae* causes disease, we sequenced the genomes of strains 168 and 168-L. This allowed us to perform the first comprehensive analysis of virulent and attenuated strains, and identify CDSs that may be related to virulence. We further investigated these putative virulence related CDSs and compared them with reported virulence determinants. Notably, almost all reported virulence determinants were found in putative virulence related CDSs. Besides the reported virulence determinants, other candidate virulence genes were also identified. The study of these candidate virulence genes and their corresponding products will be important to better comprehend the pathogenesis of *M. hyopneumonia*e.

## Results and discussion

### Genomic features of *M. hyopneumoniae* 168-L and its global comparison with pathogenic strain 168

**Figure 1:**
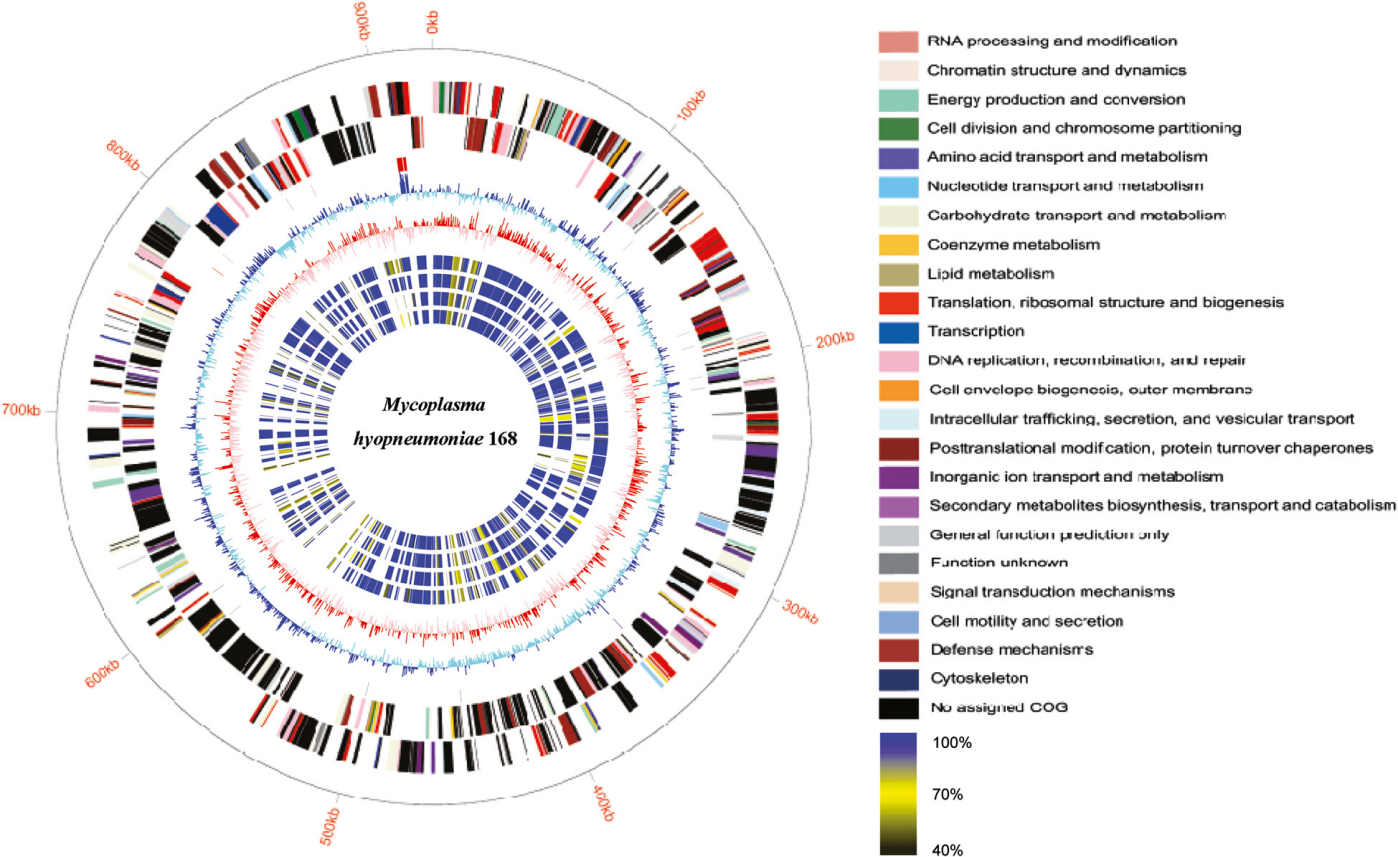
**Genome architecture.** The *dnaA* gene is at position zero. Moving inside, the first circle shows the genome length (units in *M. hyopneumoniae*); the second and the third circles show the locations of the predicted CDSs on the plus and minus strands, respectively, which were color-coded by COG categories (the color codes for the functional assignments are shown in the key); the fourth circle shows tRNAs (purple) and rRNAs (red); the fifth circle shows the centered GC (G+C) content of each CDS (blue: above mean and cyan: below mean); and the sixth circle shows the GC (G+C) skew plot (red: above zero and pink: below zero). Circles 7–10 show comparative amino acids analysis of 168 with amino acids identities color-coded according to the similarity shown in the key to strains 168-L (seventh circle), 232 (eight circle), J (ninth circle), 7448 (tenth circle).

The complete genome of *M. hyopneumoniae* 168-L consists of a 921,093 bp (GC content 28.46%) single circular chromosome (GenBank accession number CP003131). A total of 689 protein-encoding genes were predicted. The average protein size is 378 amino acids and the mean coding percentage is 84.8%. Approximately 51% of genes were assigned to specific functional clusters of orthologous groups (COGs), and 28% were assigned an enzyme classification (EC) number (Figure 1). Comparison with the *M. hyopneumoniae* 168 genome (GenBank accession CP002274) revealed a highly conserved gene content and order between the two strains. The 168-L genome is 4,483 bp smaller than that of 168 (925,576 bp), because there are 60 insertions and 43 deletions (indels; insertions and deletions of any size) in 168-L relative to 168 (see Additional file 1: Table S1; Additional file 2: Table S2; Additional file 3: Table S3). Among these, 33 indels are located in predicted CDSs, and 70 are in noncoding regions. Besides these indels, 227 single nucleotide variations (SNVs) were identified between 168 and 168-L (Additional file 4: Table S4). While 31 SNVs were mapped to intergenic regions, 196 were in coding regions, inducing amino acid substitutions, frame shifts, and translational stops.

### ISMHp1-Related genetic variations between 168 and 168-L

**Figure 2:**
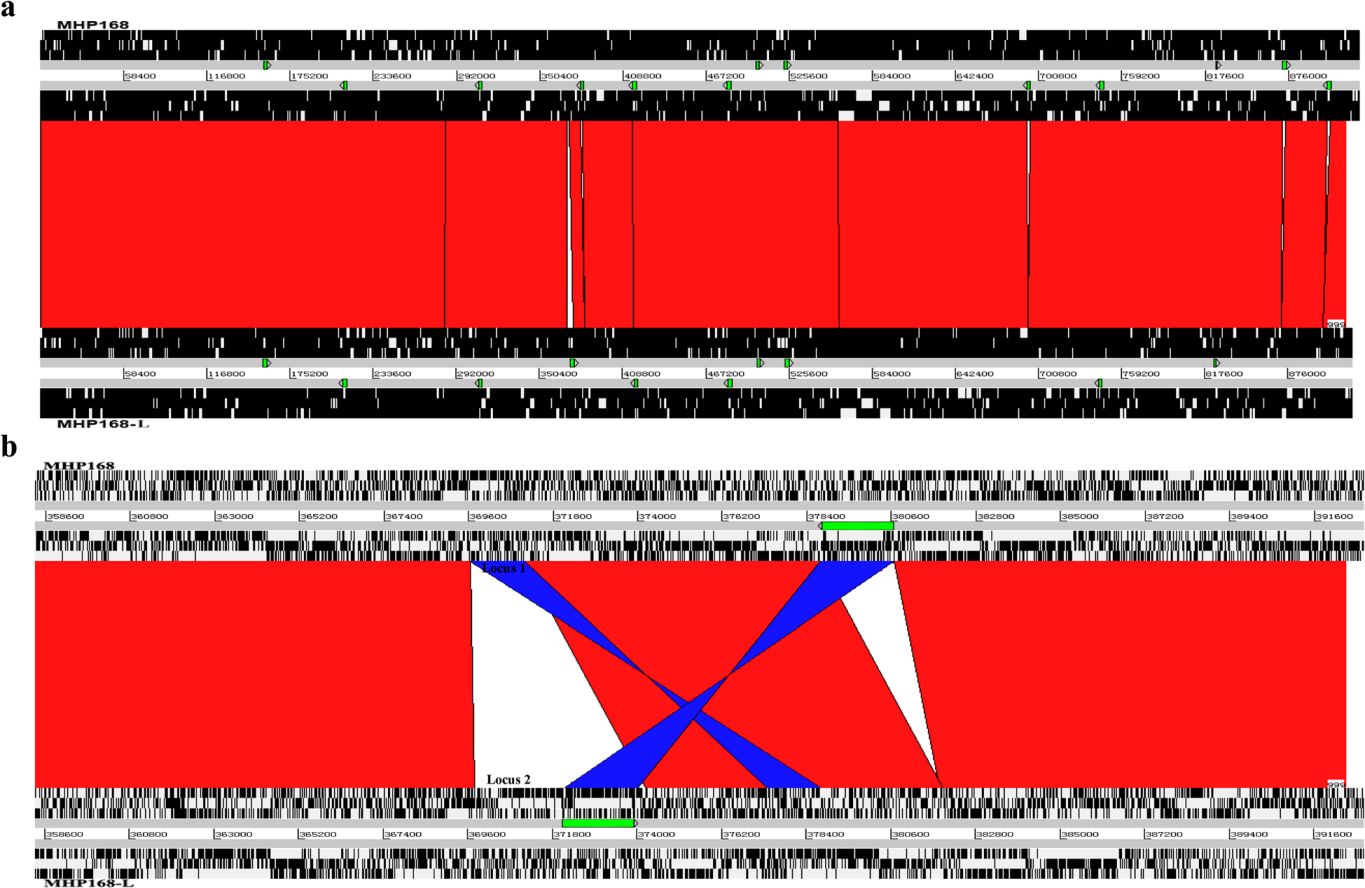
**Alignment of the whole genomes and inverted regions.** (**a**) Alignment of the *M. hyopneumoniae* 168 and 168-L genomes. (**b**) Alignment of the inverted regions of 168 relative to 168-L. The gray bars represent the forward and reverse strands. Green triangles represent ISMHP1 elements. DNA BLASTN alignments (BLASTN matches) between the two sequences are indicated by a red (same strand) or blue (opposite strand) line.

The difference between the genome sizes of strains 168 and 168-L is mainly due to differences in the duplication of Insertion Sequence (IS) elements. IS elements are distributed stochastically across the entire genome of both strains. The 168-L genome contains nine complete and one disrupted IS elements, which is almost identical to that of 168 except for slight differences in ISMHp1. There are nine complete copies of ISMHp1 in 168-L, but 12 copies in 168. The difference in ISMHp1 copy number between 168 and 168-L is due to three complete ISMHp1 deletions (located 690 kb, 870 kb, and 900 kb from *oriC*) and one complete ISMHp1 inversion, which was originally located at 378 kb from *oriC*, but was inverted in 168-L (1656 bp, located 372 kb from *oriC*) (Figure 2a).

Other than the IS elements, notable large-scale genomic differences were also indicated. Compared to strain 168, a genomic deletion of approximately 1.36 kb (locus 1: between MHP168L_311 and MHP168L_729) was identified, which had been substituted with an approximately 2.32 kb novel insertion sequence (locus 2) that was joined to a complete ISMHp1 element in 168-L (Figure 2b). This 168-L-related insertion fragment was also observed in strains 7448 and 232.

### Molecular analysis of integrative conjugative element (ICE)

The integrative conjugative element (ICE) is a mobile DNA that is probably involved in genomic recombination events and in pathogenicity. The ICEH elements are more divergent than the typical similarity of other chromosomal locus in *M. hyopneunomiae*[25], suggesting an accelerated evolution of these constins [26]. During a survey of specific sequences, a specific 26.9-kb region with similarity to the integrative conjugal element of *M. fermentans* (ICEF) [27] was found in strain 168, which was designated ICEH (for integrative conjugal element of *M. hyopneumoniae*). Unlike ICEH in strains 7448 and 232, which consist of nineteen and twenty two CDSs, respectively, the ICEH168 consist of 20 CDSs (Additional file 5: Table S5). The organization of these elements is very similar. Some CDSs present similarity to *tra* genes, which are usually associated with the bacteria conjugative plasmids such as *traK*, *traI*, *traE*[26]*.* The ICEH168 has three *tra* genes, with one *traG* and two copies of the *traE* gene. Besides, a CDS encoding for a single strand binding protein (SSB) that is essential for the transfer process is also observed.

The ICE analysis of three *M. hyopneumoniae* genomes (7448, J and 232) carried out previously, revealed that the ICEH is present in the two pathogenic strains (7448 and 232) but is absent from the non-pathogenic one (J strain) [26]. Interest has therefore shifted to questions of whether the ICEH is present in the attenuated vaccine strain 168-L. Interestingly, the ICEH was also observed in strain 168-L. Moreover, the ICEH168 and ICEH168-L are almost the same, except for a missense mutation (G192E) identified in ICEH-ORF3 (MHP168_235). Our analyses indicate that the ICEH may not only present in pathogenic strains of *M. hyopneumoniae*.

### Mutations affecting epithelium adhesion

In our previous study, the ability of adherence and damage to the cilia between strains 168 and 168-L were compared by using scanning electron microscopy. The results showed that the pathogenic strain 168 adheres to cilia inducing tangling, clumping, and longitudinal splitting of cilia, while the strain 168-L does not cause ciliary damage comparing to control group [28]. The adherence of *M. hyopneumoniae* to porcine ciliated respiratory cells is essential for the organism to colonize the respiratory epithelium and cause pneumonia [4]. The adherence process is mainly mediated by receptor-ligand interactions, and the *M. hyopneumoniae* proteins possibly involved in these interactions are obvious candidates as virulence factors [8,29-31]. We investigated the genetic variation between strains 168 and 168-L (Table 1; Additional file 6: Table S6), and compared mutations affecting CDSs corresponding to previously described mycoplasma adhesins (P97, P102, P146, P159, P216, MgPa, LppS, and LppT) [7]. Notably, almost all the reported mycoplasma adhesins are included in the CDSs affected by mutations (Table 1).

**Table 1:** Complete list of "168-L-specific" genetic variations in CDSs

**168-L gene**	**168-L gene product**	**Variation**	**168 Locus**	**Effect on 168-L coding**
MHP168L_010	Putative uncharacterized protein	SNV	MHP168_010	L368A substitution
MHP168L_014	Fructose-bisphosphate aldolase	Deletion+SNV	MHP168_014	N-terminal deletion+SNV
MHP168L_020	ABC transporter ATP-binding protein	Deletion	MHP168_020	264-aa deletion
MHP168L_022	Putative uncharacterized protein	SNV	MHP168_022	S22S substitution
MHP168L_030	Topoisomerase IV subunit A	SNV	MHP168_030	K140E substitution
MHP168L_033	Predicted protein	Deletion	MHP168_033	N-terminal deletion
MHP168L_035	hypothetical protein	SNV	MHP168_035	K177R,A221T substitution
MHP168L_058	Glycyl-tRNA synthetase	SNV	MHP168_058	P226P substitution
MHP168L_064	hypothetical protein	SNV	MHP168_064	Y552H substitution
MHP168L_065	Predicted protein	Deletion	MHP168_065	Frameshift out 20aa
MHP168L_066	hypothetical protein	Insertion+SNV	MHP168_066	Frameshift out 42aa; F324S substitution
MHP168L_069	Chaperone protein dnaK	SNV	MHP168_069	P407P substitution
MHP168L_084	Amino acid permease	SNV	MHP168_084	R13K,T396A substitution
MHP168L_085	NADH oxidase	Insertion+SNV	MHP168_085	"TG" insertion; stop; 5aa truncation; E395G substitution
MHP168L_086	Thymidine phosphorylase	SNV	MHP168_086	V15F substitution
MHP168L_103	Outer membrane protein-P95	SNV	MHP168_103	Stop;149-aa truncation
MHP168L_105	ATP-dependent protease binding protein	SNV	MHP168_105	I159I substitution
MHP168L_110	Protein p97, cilium adhesin	SNV	MHP168_110	SNV in repeat region
MHP168L_114	50S ribosomal protein L2	SNV	MHP168_114	A2A substitution
MHP168L_127	50S ribosomal protein L15	SNV	MHP168_127	E95K substitution
MHP168L_142	Phosphopentomutase	SNV	MHP168_142	N8I substitution
MHP168L_152	ribulose-phosphate 3-epimerase	Deletion+SNV	MHP168_152	I211F substitution
MHP168L_167	L-lactate dehydrogenase	SNV	MHP168_167	N204D substitution
MHP168L_168	Hexosephosphate transport protein	SNV	MHP168_168	Q122S substitution
MHP168L_182	Putative uncharacterized protein	Deletion	MHP168_182	Frameshift;105-aa truncation
MHP168L_186	Pyruvate dehydrogenase E1-alpha subunit	SNV	MHP168_186	S194G substitution
MHP168L_187	Adenine phosphoribosyltransferase	SNV	MHP168_188	534-aa N-terminal extension
MHP168L_198	Protein P102	SNV	MHP168_198	L677L substitution
MHP168L_209	ABC transporter ATP-binding protein	SNV	MHP168_209	K258E substitution
MHP168L_212	Oligopeptide transport system permease protein	SNV	MHP168_212	P203S substitution
MHP168L_235	Putative ICEF Integrative Conjugal Element-II	SNV	MHP168_235	G192E substitution
MHP168L_243	Serine hydroxymethyltransferase	Insertion	MHP168_243	No change
MHP168L_264	ISMHp1 transposase	SNV	MHP168_264	S232P,C229R substitution
MHP168L_275	lipoate-protein ligase A	SNV	MHP168_275	A179T substitution
MHP168L_284	Cobalt import ATP-binding protein cbiO 1	SNV	MHP168_284	K64E substitution
MHP168L_289	Cation-transporting P-type ATPase	SNV	MHP168_289	E100D substitution
MHP168L_308	putative ABC transporter ATP-binding protein	SNV	MHP168_308	T708A,M700L substitution
MHP168L_311	Putative uncharacterized protein	SNV	MHP168_311	D266E substitution
MHP168L_312	Putative uncharacterized protein	Insertion+SNV	MHP168_312	Insertion;R49R,I247L substitution
MHP168L_314	Putative uncharacterized protein	Insertion+deletion	MHP168_314	Stop;14-aa truncation
MHP168L_322	hypothetical protein	Insertion	MHP168_322	Frameshift;5-aa truncation
MHP168L_345	Predicted protein	Deletion	MHP168_345	Frameshift;53-aa truncation
MHP168L_355	Putative uncharacterized protein	Insertion	MHP168_355	21-aa N-terminal deletion
MHP168L_361	hypothetical protein	SNV	MHP168_361	L265L substitution
MHP168L_377	Putative uncharacterized protein	SNV	MHP168_377	E407K substitution
MHP168L_378	P60-like lipoprotein	SNV	MHP168_378	S143N substitution
MHP168L_379	HIT-like protein	SNV	MHP168_379	D58N substitution
MHP168L_381	hypothetical protein	Insertion	MHP168_381	Frameshift
MHP168L_386	P37-like ABC transporter substrate-binding lipoprotein	Insertion	MHP168_386	Frameshift out 84aa
MHP168L_389	Putative membrane lipoprotein	SNV	MHP168_389	A265S substitution
MHP168L_392	lipoprotein	SNV	MHP168_392	T6M substitution
MHP168L_394	ABC transporter permease protein	SNV	MHP168_394	A492G substitution
MHP168L_400	Ribonuclease III	SNV	MHP168_400	V58I substitution
MHP168L_401	Putative uncharacterized protein	Deletion	MHP168_401	Frameshift out 133aa
MHP168L_409	Putative type III restriction-modification system: methylase	Deletion	MHP168_409	Frameshift out 171aa
MHP168L_412	ISMHp1 transposase	SNV	MHP168_412	G50V substitution
MHP168L_413	ABC transporter ATP-binding protein	SNV	MHP168_413	D118Y substitution
MHP168L_423	Putative uncharacterized protein	SNV	MHP168_423	Y18S substitution
MHP168L_424	Lppt protein	SNV	MHP168_424	L814F substitution
MHP168L_434	hypothetical protein	SNV	MHP168_434	W107C substitution
MHP168L_444	Putative uncharacterized protein	SNV	MHP168_444	V123A substitution
MHP168L_445	Putative uncharacterized protein	Deletion	MHP168_445	3-aa deletion in repeat region
MHP168L_454	Putative uncharacterized protein	Insertion+SNV	MHP168_454	L423I substitution
MHP168L_455	Predicted protein	Deletion+SNV	MHP168_455	59-aa extension;P51P substitution
MHP168L_456	Putative uncharacterized protein	SNV	MHP168_456	substitution
MHP168L_457	Putative uncharacterized protein	Insertion+deletion+SNV	MHP168_457	Frameshift;substitution
MHP168L_462	ABC transporter ATP-binding protein	SNV	MHP168_462	V219L substitution
MHP168L_463	ABC transporter permease protein	SNV	MHP168_463	Amino acid substitution
MHP168L_473	hypothetical protein	Insertion	MHP168_473	Frameshift out 84aa
MHP168L_482	Phosphoenolpyruvate protein phosphotransferase	SNV	MHP168_482	E562G substitution
MHP168L_490	hypothetical protein	Insertion	MHP168_490	Stop;114-aa truncation
MHP168L_498	Putative uncharacterized protein	SNV	MHP168_498	D137E substitution
MHP168L_503	P216 surface protein	Insertion	MHP168_503	Q repeat insertion
MHP168L_504	P159 membrane protein	SNV	MHP168_504	G403D,L375S, G240A substitution
MHP168L_505	YX1	SNV	MHP168_505	V175I substitution
MHP168L_506	Putative uncharacterized protein	SNV	MHP168_506	N16N substitution
MHP168L_507	asparagine synthetase A	SNV	MHP168_507	I85M substitution
MHP168L_510	Oligopeptide transport system permease protein	SNV	MHP168_510	S19S substitution
MHP168L_523	Xylose ABC transporter ATP-binding protein	SNV	MHP168_523	S8S substitution
MHP168L_531	Putative uncharacterized protein	Deletion+SNV	MHP168_531	Amino acid substitution
MHP168L_541	Predicted protein	SNV	MHP168_541	L50L substitution
MHP168L_557	Potassium uptake protein	SNV	MHP168_557	D69Y substitution
MHP168L_558	Potassium uptake protein	SNV	MHP168_558	H425Y,N477D substitution
MHP168L_559	hypothetical protein	SNV	MHP168_559	D1236G substitution
MHP168L_567	hypothetical protein	SNV	MHP168_567	D68N substitution
MHP168L_571	hypothetical protein	Insertion+deletion+SNV	MHP168_571	Frameshift;amino acid substitution
MHP168L_573	Putative uncharacterized protein	Deletion+SNV	MHP168_573	Amino acid substitution;N-terminal extension
MHP168L_576	ISMHp1 transposase	SNV	MHP168_576	Amino acid substitution
MHP168L_589	Membrane nuclease, lipoprotein	SNV	MHP168_589	S98S substitution
MHP168L_596	Glycerol-3-phosphate dehydrogenase	SNV	MHP168_596	L155V substitution
MHP168L_600	ABC transporter ATP binding protein	SNV	MHP168_600	P1014L,P1037L substitution
MHP168L_606	hypothetical protein	Deletion+SNV		Frameshift;amino acid substitution
MHP168L_614	ABC transporter xylose-binding lipoprotein	SNV	MHP168_614	I51V substitution
MHP168L_621	hypothetical protein	SNV	MHP168_621	G87G substitution
MHP168L_631	ABC transporter ATP-binding-Pr1	SNV	MHP168_631	R60W substitution
MHP168L_638	Putative uncharacterized protein	Insertion+SNV	MHP168_638	K insertion in K repeat region;K7K substitution
MHP168L_639	5'-nucleotidase precursor	SNV	MHP168_639	E411K substitution
MHP168L_666	Ribosomal RNA small subunit methyltransferase G	Deletion	MHP168_666	Frameshift out 12aa
MHP168L_668	Prolipoprotein p65	SNV	MHP168_668	T138A substitution
MHP168L_671	XAA-PRO aminopeptidase	SNV	MHP168_671	G321G substitution
MHP168L_672	Putative uncharacterized protein	SNV	MHP168_672	E104E substitution
MHP168L_673	Putative uncharacterized protein	SNV	MHP168_673	I52K,E127K substitution
MHP168L_675	hypothetical protein	SNV	MHP168_675	Y175D substitution
MHP168L_676	P146 adhesin like-protein, p97 paralog	Insertion+SNV	MHP168_676	Q insertion in PQ repeat region;S404S,W404R substitution
MHP168L_688	Putative uncharacterized protein	SNV	MHP168_688	K48N substitution
MHP168L_698	hypothetical protein	SNV	MHP168_698	R126G,V159G substitution
MHP168L_707	hypothetical protein	Insertion	MHP168_707	"F" insertion
MHP168L_747	hypothetical protein	Insertion+deletion+SNV	MHP168_091	N repeat insertion;39-aa N-terminal extension;SNV
MHP168L_748	Protein P102	Deletion+SNV	MHP168_108	Stop;782-aa truncation;SNV
MHP168L_749	Putative type III restriction-modification system: methylase	Deletion	MHP168_730	N-terminal deletion
MHP168L_750	hypothetical protein	Deletion	MHP168_435	Frameshift
MHP168L_r002	16S ribosomal RNA	SNV	MHP168_r002	Amino acid substitution
MHP168L_t027	tRNA-Ser	SNV	MHP168_t027	Amino acid substitution

In 168-L, three transversions were identified in the R1 region, near the C-terminus, of P97 (MHP168_110/MHP168L_110), which encodes cilium adhesin. In *M. hyopneumoniae*, attachment to the respiratory epithelium is mainly mediated by the membrane protein P97 [4]. This protein is located on the outer membrane surface, and its role in adherence has been firmly established. To bind cilia, a minimum of eight tandem copies of the pentapeptide sequence (AAKPV/E) in R1 are required [5]. Notably, all three transversion mutations were located in the tandem repeat unit (AAKPV/E), causing an E863V substitution. Significant alteration in this critical repeat unit might partly affect the adhesion reaction in 168-L.

Previous studies have demonstrated that P102 binds fibronectin and contributes to the recruitment of plasmin(ogen) to the *M. hyopneumoniae* cell surface [15]. P102 is commonly linked to P97 cilium adhesin, forming a two-gene operon [32]. Both P97 and P102 have several paralogs within the *M. hyopneumoniae* genome. However, the paralogs have only part of the complete sequence. Interestingly, P102, the companion gene in this operon, was truncated at 564 bp by a single base insertion in strain 168. Another intact copy of P102 (99% identity) was found 85 kb from this operon. Conversely, in strain 168-L, the original truncated P102 was reverted to the intact one, while another intact copy of P102, 85 kb away, was truncated.

The P146 adhesin-like protein of *M. hyopneumoniae* shows strong similarity to the LppS lipoprotein of *Mycoplasma conjunctivae*, which is involved in *in vitro* adhesion [16]. In addition, the N-terminus region of P146 also shows strong similarity to the P97 adhesin, and has a strongly hydrophobic region (amino acids 7–29), indicating a transmembrane region, and suggesting that the protein is expressed on the surface of *M. hyopneumoniae* cells [33]. Compared with its counterpart MHP168_676 in 168, MHP168L_676 from 168-L has an in-frame insertion of one amino acid (Q) at the N-terminus of P146. The enormous intra-specific diversity shown for the P146 encoding gene is at least partly because of differences between several repeat regions present in the gene, most notably a polyserine chain of variable length, and a [Q]_n_[(P/S)Q]_m_ repeat region [34]. Interestingly, this one in-frame insertion (Q) was located in the [Q]_n_[(P/S)Q]_m_ repeat region. Polyserine chains often function as a spacer region in proteins involved in complex carbohydrate degradation [35], while sequences rich in both proline and glutamine are not uncommon and can form a conformation known as a polyproline II helix [36,37]. Such proline-rich sequences are often involved in binding processes and are highly immunogenic [37]. However, because the function of the P146 protein remains unknown, correlations with virulence or adhesion are speculative and need further investigation.

P159 is a proteolytically processed surface adhesin of *M. hyopneumoniae*[17]. Three proteins with apparent molecular masses of 27 (P27), 52 (P52), and 110 (P110) kDa were identified through proteomic analysis of *M. hyopneumoniae* lysates [17], with each representing a different region spanning P159. These cleavage fragments are located on the cell surface and present at all growth stages. In 168-L, MHP168L_504 (P159) has a missense mutation resulting in a G240A replacement in the (S)(S)G(G)S repeat region of P159. Although this (S)(S)G(G)S repeat region has been reported, its biological function is unknown.

P216 (MHP168L_503/MHP168_503) is a proteolytically processed cilium and heparin binding protein of *M. hyopneumoniae*[18]. This surface protein is post-translationally processed to generate N-terminal P120 and C-terminal P85 fragments, both of which can bind cilia [18]. The 168-L P216 gene has an in-frame four amino acid deletion in a poly Q motif near the C-terminus. Previous studies have suggested that poly Q and KEKE motifs may play a role in maintaining P85 on the cell surface [18,34]. Collectively, the deletion mutations affecting P216 may affect its cilium adhesion and may be associated with virulence attenuation in 168-L.

The MHP168L_424 gene and its gene product LppT were analyzed in detail because they showed approximately 22% identity to the LppT protein from *M. conjunctivae*. LppT is the second gene in a two gene operon with LppS, which was reported to be an adhesin in *M. conjunctivae*[16]. The LppT gene lacked a promoter and is likely to be co-transcribed with LppS, thus suggesting a functional relationship between LppS and LppT [16]. In *M. hyopneumoniae*, LppT encoded a protein of 954 aa with a calculated molecular mass 108 kDa. The gene product encoded by LppT is also a membrane protein, with a signal sequence of 34 aa at the amino-terminal end and a transmembrane structure. Notably, one of the amino acid substitutions in 168-L occurs near the C-terminus of LppT, resulting in a L814F replacement.

### Mutations altering the cell envelope and genes encoding secreted proteins

Cell envelope proteins and secreted proteins are involved in virulence, host cell interaction, and immune responses [14,38,39]. Outer membrane protein-P95 is a cell envelope protein in *M. hyopneumoniae*. In 168-L, MHP168L_103 (P95) is truncated by a nonsense mutation compared to 168, resulting in an E965* termination near the C-terminus of P95. Significant alteration in this outer membrane protein could conceivably cause a truncation in coding region, and in turn alter the function of P95 outer membrane protein.

*M. hyopneumoniae* contains an abbreviated membrane protein secretory system [1]. The pathway consists of *secA* (MHP168L_088), *secY* (MHP168L_128), *secD* (MHP168L_259), *prsA* (MHP168L_664), *dnaK* (MHP168L_069), trigger factor (MHP168L_154), and *lepA* (MHP168L_076). It has recently been demonstrated that some pathogenic bacteria use a type IV secretion system, composed of subunits related to the conjugation machinery, to deliver effector molecules to host cells [40], and that this system may be involved in pathogenesis [41]. We found no pathogenic mutations in the protein secretory system, except a synonymous substitution (P407P) in MHP168L_069, which encodes chaperone protein *DnaK*.

### Mutations affecting antigens

Studies on the antigenic properties of *M. hyopneumoniae* revealed several immunodominant proteins, including the P36 cytosolic protein [12,42], the P46, P65, and P74 membranous proteins [43-46], the elongation factor Tu [47], the chaperone protein *DnaK*[47], the pyruvate dehydrogenase E1-beta subunit [47], and the P97 adhesin [4]. The functions of these proteins have not been well elucidated, but specific reactants may eventually be useful tools to diagnose *M. hyopneumoniae*[48].

The cytosolic P36 protein is a lactate dehydrogenase [49] that induced an early immune response in pigs that are experimentally and naturally infected by *M. hyopneumoniae*[50]. Comparative studies with other *Mycoplasmas* commonly found in pigs demonstrated that the P36 proteins carry highly conserved species-specific antigenic determinants for *M. hyopneumoniae*[42]. Hyperimmune sera produced against recombinant P36 protein showed no reactivity against other porcine *Mycoplasmas*, including *M. flocculare*, *M. hyorhinis*, and *Acholeplasma laidlawii*[12]. Notably, one of the observed amino acid substitutions in 168-L occurs near the C-terminus of P36 (MHP168L_167), resulting in a N204D replacement.

P65 is an immunodominant surface lipoprotein of *M. hyopneumoniae* that is specifically recognized during disease [14]. Analysis of the translated amino acid sequence of the gene encoding p65 revealed similarity to the GDSL family of lipolytic enzymes [14]. The monospecific antibodies against heat shock protein-like P42 antigen, part of P65, can block the growth of *M. hyopneumoniae*[51]. In 168-L, MHP168L_668 (P65) has a missense mutation resulting in a T138A replacement.

### Mutations affecting transport proteins

As *Mycoplasmas* are dependent on the exogenous supply of many nutrients, it has been predicted that they may need many transport systems [3]. Motif analysis revealed a family of proteins with a phosphotransferase (PTS) motif. The open reading frames (ORFs) included *sgaA* (MHP168L_422), *sgaB* (MHP168L_421), *sgaT* (MHP168L_563), *mtlF* (MHP168L_739), *mtlA* (MHP168L_561), *nagE* (MHP168L_582), and *licA* (MHP168L_041) [7]. However, no mutations were identified in this PTS transporter family. There are approximately 30 genes with ABC transporter family signatures in the genome of *M. hyopneumoniae* 168-L (Additional file 7: Table S7), and five missense mutations and one synonymous substitution were identified in this group. These included a cobalt import ATP-binding protein (MHP168L_284, K64E), an ABC transporter permease protein (MHP168L_394, A492G), a xylose ABC transporter ATP-binding protein (MHP168L_523, S8S), and three ABC transporter ATP-binding proteins (MHP168L_413, D118Y; MHP168L_462, V219L; MHP168L_631, R60W). Interestingly, the expression of MHP168L_394 and MHP168L_413 was reported to be up-regulated *in vivo* during disease relative to *in vitro*-grown [11]. The variability between strains 168 and 168-L in multi-transport proteins indicates that they may affect growth and survival in different hosts or host tissues.

### Mutations affecting genes directly related to metabolism and *in vivo* growth

**Figure 3:**
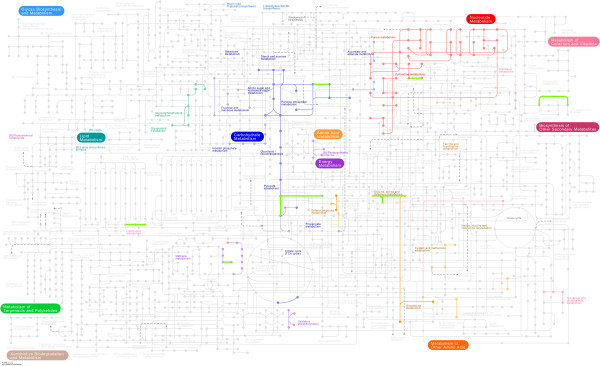
**Metabolic potential.** The metabolic pathways of *M. hyopneumoniae* strains 168 and 168-L were mapped and analyzed using KEGG Pathway Database. Those pathways, containing mutations affected metabolic-related genes, are shown in green.

*M. hyopneumoniae* strain 168 encodes 695 genes, approximately one quarter of which are involved in metabolism and *in vivo* growth. Of particular interest were the mutations observed in the genes involved in various metabolic pathways (Figure 3), including glycolysis/gluconeogenesis (MHP168_167, MHP168_186), purine metabolism (MHP168_289, MHP168_639), pyrimidine metabolism (MHP168_086), glycerophospholipid metabolism (MHP168_596), oxidative phosphorylation (MHP168_085), aminoacyl-tRNA biosynthesis (MHP168_058), and the pentose phosphate pathway (MHP168_142, MHP168_152). An in-frame insertion of two amino acids (TG), a missense mutation (E393G) and a nonsense mutation (N456*) were identified in 168-L near the C-terminus of MHP168L_085, which encodes a NADH oxidase involved in oxidative phosphorylation. *Mycoplasma* genomes are deficient in genes coding for components of intermediary and energy metabolism [3]. Thus, *Mycoplasmas* depend mostly on glycolysis to synthesize ATP [3]. In the glycolysis pathway, missense mutations in both L-lactate and pyruvate dehydrogenase were observed, resulting in N204D and S194G replacements, respectively. Iron deprivation, is a prominent feature of the host innate immune response, and most certainly impacts growth of *Mycoplasmas in vivo*[52]. Through transcriptome analysis, MHP168_639 was identified to be down-regulated during iron limiting conditions [52]. This suggests that MHP168_639 may play a role in *M. hyopneumoniae*’s response to iron stress. In 168-L, MHP168L_639 has a missense mutation resulting in an E411K replacement. Mutations in these metabolism-related genes accumulated over 300 *in vitro* passages likely affect growth and survival within host cells.

In addition, approximately 41% of mutations affected genes coding for hypothetical proteins. Despite the lack of functional annotations for these genes, their disruption in 168-L makes them obvious targets for investigation as potential virulence factors. Further molecular genetics and *in vivo* studies are required to confirm and assess the relative importance of these genes in the attenuation of virulence in 168-L.

## Conclusions

We successfully used a combination of sequencing genomics and comparative genomics strategies to provide a comprehensive analysis of virulent and attenuated *M. hyopneumoniae* strains to identify determinants involved in pathogenesis. The genome of the attenuated high-passage derivative strain 168-L was sequenced and compared to virulent strain 168, revealing mutations in numerous CDSs. These mutations affected CDSs are likely to be associated with virulence. We then compared these putative virulence factor CDSs to reported virulence determinants. Notably, almost all of the reported *M. hyopneumoniae* virulence determinants were included in the list of putative virulence factor CDSs. Variations in the previously described mycoplasma adhesins (P97, P102, P146, P159, P216 and LppT), cell envelope proteins (P95), cell surface antigens (P36), secreted proteins, chaperone protein (*DnaK*), and genes directly related to metabolism and *in vivo* growth may contribute to loss of virulence in 168-L. We then proceeded to characterize the alterations in gene functions caused by mutations at the protein level, and compared those mutations with previously described repeat motifs that may be of primary importance for virulence [34]. Interestingly, we found that many mutations were located in the virulence associated motifs of the various proteins. To bind cilia, a minimum of eight tandem copies of the pentapeptide sequence (AAKPV/E) in the R1 region of P97 are required [5]. We identified three mutations in the tandem repeat unit (AAKPV/E), causing an E863V substitution. A similar situation was also observed in several other virulence associated genes (P146, P159, P216, and LppT). We hypothesize that the cumulative effect of mutations in virulence associated genes may account for the attenuation of virulence in 168-L. In this study, a total of 330 genetic variations were identified. While these included reported virulence-related genes, other novel virulence determinants were also identified. However, further molecular genetics and *in vivo* studies are required to confirm and assess the relative importance of these suspected novel virulence determinants in the attenuation of virulence. The comparative genomic analysis presented here will not only provide insights into the basis of attenuation of virulence in 168-L, but may also provide targets for mutagenesis in the pursuit of development of a more efficacious vaccine.

## Methods

### Bacterial strains, growth conditions, and DNA extraction

Clonal isolates of *M. hyopneumoniae* strain 168 and 168-L were selected for sequencing. Both of the strains were grown in KM2 cell-free medium at 37°C. The culture was harvested from 100 mL KM2 cell-free medium by centrifugation at 1,200×*g* for 30 min, and then total genomic DNA was extracted from mycoplasma cultures using a TIANamp Bacteria DNA Kit (Tiangen, Beijing, China) according to the manufacturer’s instructions.

### Genome sequencing and assembly

Genomic libraries containing 8 kb inserts were constructed according to the manufacturer’s protocols. Whole-genome sequencing of strain 168-L was performed by combining GS FLX and Solexa paired-end sequencing technologies. A total of 242,507 reads (67.4% paired ends) were produced with the GS FLX system, giving 44.5-fold coverage of the genome. Eighty-eight percent (215,346) of reads were assembled into one large scaffold using Newbler (454 Life Sciences, Branford, CT, USA). A total of 1,971,358 reads were generated with an Illumina Solexa genome analyzer IIx (Illumina, San Diego, CA, USA) and were mapped to the scaffold with the Burrows-Wheeler Alignment (BWA) tool [53]. Gaps were filled by local assembly of the Solexa/Roche 454 reads or sequencing PCR products using a Prism 3730 capillary sequencer (Applied Biosystems, Foster City, CA, USA). All repeated DNA regions and low-quality regions were verified by PCR and sequencing of the product amplified from genomic DNA.

### Annotation and sequence analyses

Open reading frames containing more than 30 amino acid residues were predicted using Glimmer 3.0 [54] with modified genetic code 4 and verified manually using the strain 168 annotation. Loci discrepancies between the 168 and 168-L consensus sequences were manually examined for support at the trace data level. Transfer RNA (tRNA) and ribosomal RNA (rRNA) genes were predicted using the tRNAscan-SE program [55] or by observing similarities with the *M. hyopneumoniae* strain 232 and strain J rRNA genes. Artemis (release 12) [56] was used to collate and annotate data. Functional predictions were based on BLASTP similarity searches against the UniProtKB [57], GenBank [58], Swiss-Prot protein [59], and COG [60] databases. EC numbers were assigned using the Kyoto Encyclopedia of Genes and Genomes (KEGG) [67] and metabolic pathways were mapped and analyzed using KEGG Pathway Database (http://www.genome.jp/kegg/pathway.html). Pseudogenes were detected by BLASTN analysis, comparing the genome sequences of 168-L with those of 232 and J, and then the annotation was revised manually.

### Single nucleotide polymorphism (SNP) analysis

Nucleotide comparisons and single nucleotide polymorphism (SNP) analysis for strains 168 and 168-L were performed using the Artemis Comparison Tool (ACT) [61] and Mauve 2.3.1 genome alignment software [62]. ORF graphical visualization and manual annotation were carried out using Artemis, release 12 [56]. Screening for unusual coding differences between the 168 and 168-L genomes (stops and frame shifts) was conducted using FASTA program packages [63,64] and BLAST [65]. The coding differences between the 168 and 168-L genomes were checked manually.

### Accession numbers

*M. hyopneumoniae* strains 168 and 168-L genome sequences have been deposited in GenBank under accession numbers CP002274.1 and CP003131, respectively.

## Abbreviations

EP: Porcine Enzootic Pneumonia; SNVs: Single Nucleotide Variations; MPS: Mycoplasmal Pneumonia Of Swine; COG: Clusters Of Orthologous Groups; EC: Enzyme Classification; IS: Insertion Sequence; ICE: Integrative Conjugative Element; SSB: Single Strand Binding Protein; PTS: Phosphotransferase; BWA: Burrows-Wheeler Alignment; tRNA: Transfer RNA; rRNA: ribosomal RNA; KEGG: Kyoto Encyclopedia of Genes and Genomes; ACT: Artemis Comparison Tool.

## Competing interests

The authors declare that they have no competing interests.

## Authors’ contributions

Conceived and designed the experiments: WL SX LF HC. Performed the experiments: WL ML SL SG ZF. Analyzed the data: WL ZZ GS RL BL. Contributed reagents/materials/analysis tools: WL GS RL BL. Wrote the paper: WL LF SX. All authors read and approved the final manuscript.

## Supplementary Material

Additional file 1: Table S1 Nucleotide differences identified between genomes of 168 and 168-L. Click here for file

Additional file 2:Table S2 Insertions detected in 168-L compared to 168. Click here for file

Additional file 3: Table S3 Deletions detected in 168-L compared to 168. Click here for file

Additional file 4: Table S4 SNVs detected in 168-L compared to 168. Click here for file

Additional file 5: Table S5 Annotated CDS of the elements ICEH168 and ICEH168-L. Click here for file

Additional file 6: Table S6 Complete list of "168-L-specific" genetic variations in intergenic. Click here for file

Additional file 7: Table S7 Mutations affecting genes with ABC transporter family signatures. Click here for file
